# NEFI: Network Extraction From Images

**DOI:** 10.1038/srep15669

**Published:** 2015-11-02

**Authors:** M. Dirnberger, T. Kehl, A. Neumann

**Affiliations:** 1 Max Planck Institute for Informatics, 66125 Saarbrücken, Germany

## Abstract

Networks are amongst the central building blocks of many systems. Given a graph of a network, methods from graph theory enable a precise investigation of its properties. Software for the analysis of graphs is widely available and has been applied to study various types of networks. In some applications, graph acquisition is relatively simple. However, for many networks data collection relies on images where graph extraction requires domain-specific solutions. Here we introduce NEFI, a tool that extracts graphs from images of networks originating in various domains. Regarding previous work on graph extraction, theoretical results are fully accessible only to an expert audience and ready-to-use implementations for non-experts are rarely available or insufficiently documented. NEFI provides a novel platform allowing practitioners to easily extract graphs from images by combining basic tools from image processing, computer vision and graph theory. Thus, NEFI constitutes an alternative to tedious manual graph extraction and special purpose tools. We anticipate NEFI to enable time-efficient collection of large datasets. The analysis of these novel datasets may open up the possibility to gain new insights into the structure and function of various networks. NEFI is open source and available at http://nefi.mpi-inf.mpg.de.

The study of complex network-like objects is of increasing importance for many scientific domains. The mathematical study of networks, Graph Theory, formalizes a network’s structure by modeling the constituents of a network as *vertices* and the pairwise relations between them as *edges*. Some communities traditionally refer to vertices as nodes or sites and to edges as arcs or links. Networks are ubiquitous in everyday life. Examples are as diverse as the Internet, social networks, transportation networks, metabolic networks, blood vessels or the vein networks of leaves. For a comprehensive review see^1^.

In situations where the extraction of a mathematical graph from a physical network is easy, the size of graphs that can be analyzed quickly increased from hundreds to millions of vertices. At the same time it became feasible to build large databases of various types of networks. This enabled the application of software incorporating methods from statistics and graph theory to obtain many results that changed our understanding of large scale network structures. However, digitization remains difficult for many types of networks, e.g. leaf venations, blood vessels or food webs, and therefore ready-to-analyze datasets are often not available. In these cases, investigation on a larger scale requires tedious and sometimes error prone data acquisition.

In many experimental settings networks are initially available as high quality images obtained under laboratory control. Before any analysis can take place, it is necessary to extract the associated graphs from these images. This requires the identification of vertices and edges within the depicted structure. This process can quickly become very work-intensive even for smaller networks, which makes automated solutions indispensable.

Leveraging advances in computer vision, several authors have proposed and successfully implemented solutions for domain specific graph extraction applications. The authors of^2^,^3^ consider the mycelial networks of *P. impudicus*. They use watershed segmentation in combination with a novel enhancement step designed to highlight curvilinear features in the input networks. Based on the segmented image a skeleton is computed and used to extract the graph representing the input network. The resulting method is designed to be brightness and contrast invariant in order to correctly extract the networks grown by *P. impudicus* from challenging noisy or low contrast images.

Baumgarten *et al.*^4^,^5^ investigate the vein networks of *P. polycephalum*. For segmenting the input image they rely on careful constant thresholding followed by a sequence of restoration algorithms that try to repair the network in the segmented image. Next, the restored segmented image is used to compute a skeleton. After applying another sequence of correction steps, the skeleton is scanned to extract the graph of the input network.

In^6^ a more general algorithm applicable to a variety of problems is proposed. Based on an original stochastic model, the authors use Monte Carlo sampling to obtain junction-points in the input image. This technically involved solution guarantees structural coherence for the resulting graph representation. Further examples include the extraction of road networks^7^, retinal blood vessel analysis^8^ and the extraction of plane graphs^9^.

The three above mentioned algorithmic solutions for the network extraction problem exhibit one or more of the following limitations:

- They do not build on top of well-established computer vision methods and tend to rely on ad-hoc algorithms. As a result the quality of the method and its implementation could likely be improved. In addition, a lot of time is spent on reimplementing algorithms that are already available.
- They are not implemented or only available as pseudo-code.
- They are implemented but not designed for easy of use, distribution and extendability.

We are aware that the primary objective of the work cited above is not the production of reusable software, but of algorithms and tools for solving a concrete research question. As a result, the above authors have limited time for researching advances in computer vision, following best software engineering practice or writing documentation respectively.

From experience we know that when producing an easy-to-use software, a large part of the required work consists of specifying and improving the user-interface as well as working out minor bugs and annoyances. This type of work, while time consuming, is essential for any software aiming to reach a non-negligible audience. However, efforts like these are hardly attractive to researchers whose focus is on obtaining the next result. While we understand that under these circumstances the aforementioned limitations arise naturally, we strongly believe that it is necessary to overcome those limitations in order to increase the value and the impact of scientific software in general and network extraction software in particular.

To this end, we introduce NEFI, a lightweight piece of ready-to-go software intended to enable the non-expert to automatically extract networks from images. NEFI constitutes an extensible framework of interchangeable algorithms accessible through an intuitive graphical user interface.

We emphasize at this point that we do not claim to introduce novel techniques for image processing or computer vision. Instead, our contribution consists of a reusable, flexible and easily extendable toolbox combining well-known methods, which have become standard in their respective fields of origin, in a meaningful way. By introducing NEFI, we hope to make these methods more widely accessible to practitioners in other fields.

NEFI’s segmentation is based on a combination of standard routines available in OpenCV, (2015)^10^, These algorithms are known to perform well on clean and uncluttered images obtained under controlled laboratory conditions. However, on more challenging inputs of low contrast, strong gradients or similar irregularities, their performance is severely reduced. Nevertheless, in these cases more involved algorithms, currently not implemented as part of a reliable library and thus not integrated into NEFI, may still be able to process these images. To help meet this situation, NEFI was designed with extendability in mind. As a result users will find it easy to build on-top of NEFI’s code in order to add their own implementations of more sophisticated methods.

## Network Extraction From Images

NEFI features a collection of image processing routines, segmentation methods and graph algorithms designed to process 2D digital images of various networks and network-like structures. Its main function is executing a so-called extraction pipeline, designed to analyze the structures depicted in the input image. An extraction pipeline, for short pipeline, denotes an ordered sequence of algorithms. A successful execution will return a representation of the network in terms of an edge-weighted undirected planar graph. Computed weights include edge lengths and edge widths. Once the graph is obtained, available graph analysis software^11^,^12^,^13^,^14^,^15^,^16^ or custom written scripts can be deployed to investigate its properties.

**Figure 1:**
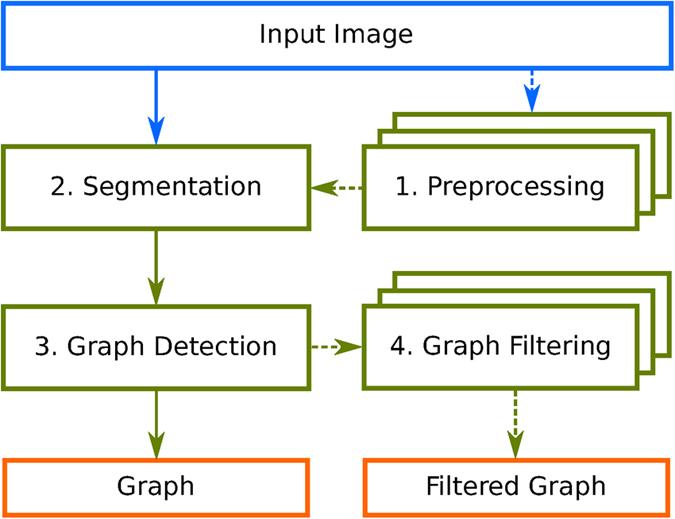
A flow chart illustrating NEFI’s pipeline components in green boxes. Dashed arrows depict optional sections of the pipeline. Blue and orange boxes denote NEFI’s input and possible outputs respectively.

**Figure 2:**
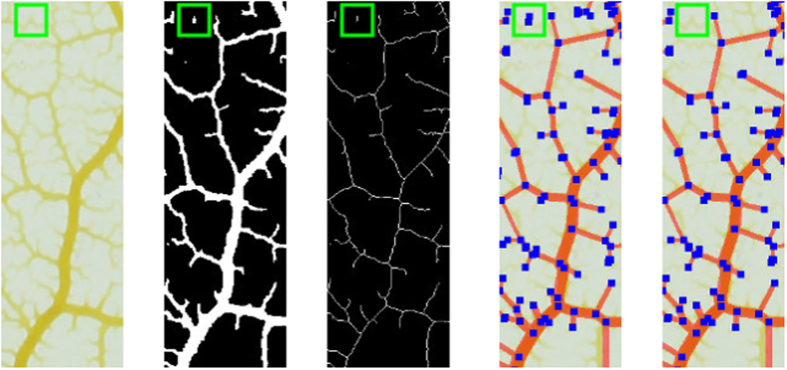
Direct comparison of NEFI’s pipeline steps given a slice of an image of a slime mold (*Physarum polycephalum*). From left to right: input image, segmented image, skeletonized image, detected graph and filtered graph. The green square contains a very faint vein which the segmentation did not pick up fully. Thus, the skeleton becomes fragmented which leads to spurious vertices in the detected graph. By applying a graph filter we remove stray vertices without manipulation of the segmented or the skeletonized image. Similar filtering can remove “dead-ends”, i.e. vertices that do not belong to any cycle in the graph.

A typical pipeline combines algorithms from up to four different classes: preprocessing, segmentation, graph detection and graph filtering, see Fig. 1. A more detailed description follows below. For each pipeline section, NEFI typically offers several interchangeable algorithms to choose from. After executing preprocessing routines, a segmentation algorithm separates foreground from background. Then the foreground is thinned to a skeleton from which the vertices and edges of the graph are determined. In the process various edge weights are computed. Finally, the graph can be subjected to a variety of useful graph filters. Figure 2 illustrates the intermediate results of NEFI’s pipeline steps listed in the order of their execution. When a pipeline is executed, NEFI makes all intermediate results available via its clean and intuitive GUI, see Supplementary Fig. S7.

Using the GUI all basic functions of NEFI can be accessed in an intuitive fashion. To facilitate ease-of-use, most of NEFI’s algorithms come with default parameters based on the settings in OpenCV, (2015)^10^, which were found to perform well on our test sets as well as on many other images.

There are various predefined pipelines to get started immediately. Alternatively, users may freely combine the various methods to build custom pipelines. Both approaches allow the user to experiment with the available methods in order to close in on the optimal settings for the data. Once a pipeline is constructed, it can be saved and reused. NEFI’s simple pipeline concept together with a self-explanatory graphical user interface make working with NEFI intuitive and straightforward. NEFI also offers a command-line mode, which is suited for batch processing.

**Figure 3:**
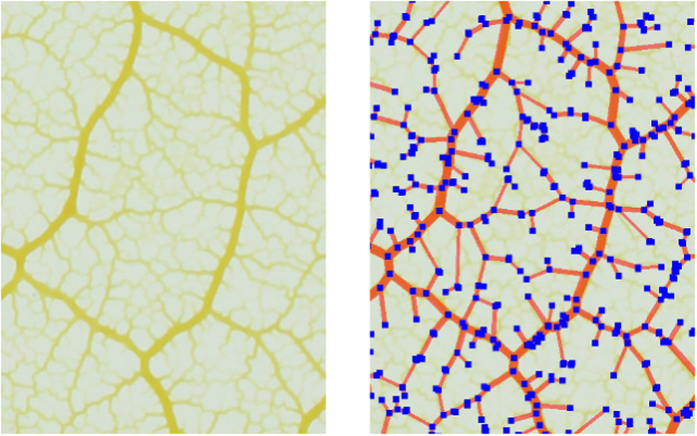
Extracted graph of the network formed by a slime mold (*Physarum polycephalum*). The left hand side shows the input image depicting the network. The right hand side shows the extracted graph overlayed on top off the same image for direct comparison. Note, that no filters have been applied. The image was produced in a collaboration with the KIST Europe.

**Figure 4:**
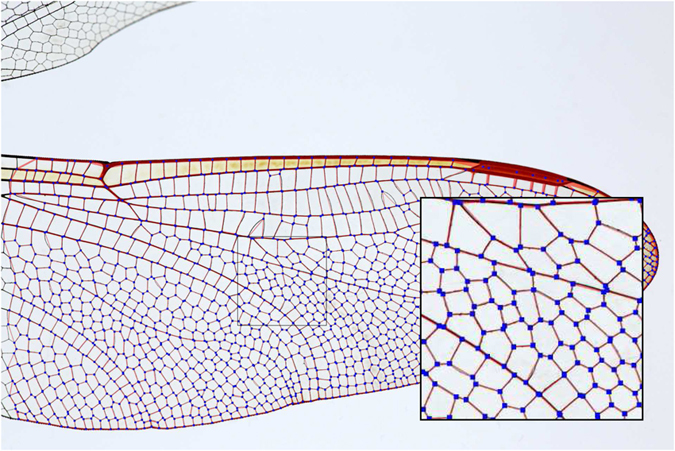
Extracted graph of the vein network exhibited by a wing of a dragonfly (*Ajax junius*). Note, that after the use of various filters a very clean-looking graph is obtained. Image courtesy of Pam and Richard Winegar.

NEFI comes with a number of example images from different domains which we use to produce the figures in this work. Figures 3 and 4 show NEFI’s output on two images using predefined pipelines. Blue squares denote the vertices and red lines the edges of the detected graph. The thickness of the detected edges corresponds to thickness of the depicted structures. For comparison the extracted graph is drawn on top of the input image. We present a detailed quantitative evaluation in a later section.

We stress that NEFI can deal with a range of inputs from various domains as long as they are of sufficient quality. In addition to the examples shown above, it has been successfully used to process images of natural (e.g. leaf venation, patterns of mud cracks) as well as man-made structures (tilings). It is also straightforward to add custom extensions. We provide a well documented platform which allows programmers to include more specialized segmentation algorithms or additional graph filters. For an overview of alternative graph extraction approaches see for example^17^.

Next, we discuss the purpose and design of each major stage of the pipeline and highlight some of NEFI’s strong points.

## Preprocessing Collection

The preprocessing section of the pipeline offers various standard image processing algorithms intended to be used prior to the segmentation step. Preprocessing methods may be exploited to affect the output of the segmentation step. For example, adding a slight blur to an input image may benefit the overall result by reducing the amount of spurious white pixels in the segmented image. However, blurring too much will remove fine detail and reduce accuracy in determining the thickness of depicted edges. As a result, we recommend to experiment with different approaches and parameter settings in order to decide how to use preprocessing. For images of sufficient quality we found that excellent results can be obtained without preprocessing.

NEFI relies on OpenCV, (2015)^10^, for preprocessing and offers Gaussian and Median Blurring, Denoising as well as Bilateral Filtering.

## Segmentation Collection

The goal of the segmentation step is separating the image foreground, i.e. the structures of interest, from the remaining image. NEFI builds on top of OpenCV, (2015)^10^, combining different segmentation algorithms. The general-purpose algorithms shipped with NEFI have become standard in image processing and perform reliably well if input images are clean, devoid of strong gradients and have a good contrast between fore- and background. Conversely, if the input becomes more challenging, the effectiveness of NEFI’s segmentation degrades quickly and more complex or domain-specific algorithms become necessary. We defer a quantitative study of how the properties of the input image affect NEFI’s performance to the Section *Evaluation*.

NEFI’s segmentation is designed such that several algorithms can be used interchangeably. We included basic thresholding algorithms like Otsu’s method^18^ or adaptive thresholding as well as more involved segmentation routines such as guided watershed^19^ and the GrabCut algorithm^20^. The last two methods receive as an additional input a so-called marker. The better the markers approximate the foreground, the better these algorithms work. NEFI offers several marker strategies which can be used interchangeably together with the respective marker based segmentation routines.

Interchangeability of the algorithms is a core design principle of all pipeline steps.

This design facilitates easy experimentation with different methods. Our own experience shows that often it is not clear a priori which methods work for a given input image. This decision usually also depends on the desired degree of detail in the final output, where less sensitive methods might produce fewer false positives. This ease of experimentation with quick visual feedback from the GUI is one of the major strong points of NEFI.

The flexibility is not limited to the algorithms we provide. Instead, NEFI’s software design makes it easy to integrate additional methods. We expect that in practice challenging inputs will be encountered for which the algorithms currently offered by NEFI will be insufficient. In these cases a potential user may choose to implement additional, perhaps domain-specific, methods. By extending NEFI, the user can rely on existing modules and thus save a lot of time. The authors are convinced that improved extendability is another strong point of our work.

## Graph Detection Collection

The graph detection collection consists of algorithms that take a segmented image as input and detect the nodes and the edges of the graph. We offer a colloquial description of the actual algorithm because we do not rely on well-documented library code for this section of the pipeline.

The first step for graph detection is called thinning. Here we reduce the segmented foreground such that every line is only one pixel thick, while preserving the connectivity properties of both the foreground and the background pixels. The result of this process is called the skeleton of the segmented image. To do so we implemented the algorithm by Guo and Hall^21^. It always produces thin results and preserves 8-connectivity of the foreground pixels. A pure Python implementation proved to be fairly slow, hence we chose to implement this function as a C extension.

For fairly thin foreground features this method is nearly flawless and finds a skeleton where the lines lie in the center of the foreground areas. However, large foreground sections lead to artifacts in the skeleton whose exact shape depends on the noise present at their borders.

On the skeleton we then detect the positions of nodes. For this purpose we adapt criteria from thinning algorithm by Zhang and Suen^22^. A white pixel becomes a node if its removal creates exactly one or at least three 4-connected white components in its 1-neighborhood. In the former case the pixel forms the end of a path, otherwise it is the meeting point of at least three edges.

Note that due to this step, the maximum degree of the graphs we detect is limited to four. This is inevitable if nodes are detected at single-pixel locations. For higher degree nodes we will create several nodes of limited degree that are very close to each other and can be merged by a later post-processing step.

Given the node positions, it is very simple to find the edges. We perform a variant of breadth first search on the white pixels in the skeleton, starting from each node simultaneously. Each white pixel around a node gets a unique number and a queue. In each step we iterate over all queues and take out the first pixel. If it is unmarked, we mark it with the unique number of this queue and enqueue all its white neighbors. Otherwise, we have detected an edge, i.e. there is a path along white pixels that connects two nodes.

While walking along the pixels we record the length of the edge. Horizontal and vertical steps count as one unit, diagonal steps count as 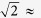 1.41 units.

The diameter of an edge calculated by computing a distance transform on the segmented image. This assumes that the thinned edge lies in the middle of the actual edge. Computing the diameters is now a simple lookup of each edge-pixel from the skeleton in the distance transformed image. As we have the diameters along the whole edge on hand by this procedure, we then compute a median and a variance.

For handling the graph we rely on NetworkX, (2014)^23^.

## Graph Filter Collection

The graph filter collection offers the possibility to add powerful processing steps that directly apply to the graph obtained after graph detection.

Often it is possible to improve the result by removing unwanted artifacts in the segmented image or during later processing stages. A common strategy, used for example in^4^,^5^, consists of “repairing” the errors in the skeleton using heuristics or user assisted methods. However, these methods carry the potential danger of introducing additional errors.

NEFI pursues a novel approach by exploiting the structure of the extracted graph. First, we retain a maximum of structural information by not altering the segmented image or the skeleton at all, i.e. we establish the graph including all artifacts. Then we use dedicated graph filters to remove said artifacts. For example, if the network in the input image is reasonably large, it will result in a large connected component in the graph. Small components resulting from noise can thus be removed effectively and safely. Since the effects of filtering the graph can immediately be evaluated by visual inspection, we prefer graph filtering over less transparent approaches that take place before the graph was established. Figure 2 illustrates the use of filtering.

We have used filtering with sensitive segmentation to obtain surprisingly good results. Overly sensitive segmentation picks up fine detail but also introduces artifacts. However, almost all of the artifacts result in very small components that can easily be removed by filtering. The desired detail will remain mostly unaffected because it is part of the largest component. The graph depicted in Fig. 4 was obtained using this technique.

Filtering may also be used to remove parts of the graph which are not of interest. The following filters are predefined in NEFI. A filter removing everything not in the largest connected component, one smoothing vertices of degree two except if this introduces parallel edges and finally, one which removes all vertices and edges that are not contained in a cycle. Filters may be freely combined in any order. Naturally, the filter collection is designed for extendability.

Graph filtering and its various applications delivers excellent results. To our knowledge, no other software offers such tools as part of its core workflow. For this reason, we consider this one of NEFI’s strong points.

## Evaluation

To assess NEFI’s performance we investigate the output quality given input images of varying quality. Additionally we report on NEFI’s speed.

## Defining a Graph Similarity Measure

As a quality measure, we need to quantify the degree of congruence between the graph depicted in the original input image *i* and the graph computed by NEFI.

Let 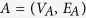 be the *true* graph correctly describing the structure depicted in *i*, with *V*_*A*_ and *E*_*A*_ denoting its vertex and edge set respectively. We call *A* the *ground truth*, which is of course not known in general. Furthermore, let *B* denote the graph obtained by executing one of NEFI’s pipelines. Note that, 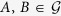, where 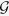 denotes the set of undirected edge-weighted planar graphs where vertices are labeled with their respective euclidean coordinates in the plane. With these definitions we propose a similarity measure *s* mapping any pair of graphs 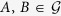 onto a number 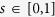.

We compute a correspondence of vertices in *A* to vertices in *B*. Two edges 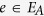 and 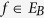 then correspond if their endpoints correspond. We choose the correspondence such that the following intuitive notion of similarity are optimized.

1. Positions of vertices in *V*_*A*_ are similar to positions of corresponding vertices in *V*_*B*_.
2. Edges in *E*_*A*_ including their weights are similar corresponding edges in *E*_*B*_.

For an exact definition of *s* and the notions of *similarity* and *correspondence*, we refer the reader to the supplementary material.

We require that the measure is maximal if any graph *A* is compared with itself, that is 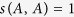. Consequently, if *A* is completely different from *B* we have 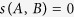. This minimum value is assumed if no viable correspondence between *V*_*A*_ and *V*_*B*_ can be found. Naturally, the value of *s*(*A*, *B*) increases (decreases) if the similarity between *A* and *B* increases (decreases).

## Evaluation of NEFI’s Output

We proceed with the evaluation of NEFI’s output using the above similarity measure. To do so we create a set 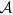 of ground truth graphs such that 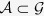. We start by processing a real-life set *I*_0_ of images of the slime mold *P. polycephalum* with NEFI. Thus we obtain a set of graphs 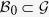. Given those graphs we obtain the set 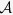 by distorting the graphs 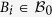 using different graph filters. At this point we will not use the images in *I*_0_ or the graphs in 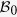 anymore.

Next, we turn the graphs in 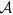 into a test set *I*_1_ of 2D images by simply drawing them. The drawing preserves the euclidean positions of the nodes, the edge lengths and the thickness of the edges. As a result, the image 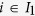 depicts the graph 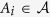. In other words, we know the *ground truth A*_*i*_ for every image *i* in the test set *I*_1_.

To compare different segmentation methods, we prepare a set 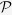 of pipelines differing only in the segmentation algorithms used. The parameters of the pipeline where chosen manually for each test set using experimentation and visual inspection.

Given the sets *I*_1_ and 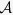 as well as our similarity measure we can now evaluate NEFI’s output. We take an image 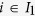 and process it with a given 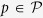 to obtain a graph 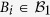, i.e. the graph NEFI extracted from the input image. Then we compute the similarity 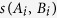. To obtain statistical statements, we repeat this procedure for all images and all pipelines.

During the computation of 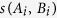, we record features NEFI failed to detect in 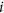, namely the number of vertices (edges) in *A*_*i*_ which remain without corresponding vertices (edges) in *B*_*i*_. This is the number of false negatives (FN). Furthermore, we record the number of vertices (edges) in *B* for which no corresponding vertices (edges) exist in *A*_*i*_. These are features which NEFI detects in *i* but which are in fact not present. These are false positives (FP). Finally we record the number of vertices (edges) in *B*_*i*_ that have corresponding elements in *A*_*i*_. That is, features correctly extracted from the image *i*, which we count as true positives (TP). Unfortunately, we cannot determine the number of true negatives (TN) in a similar fashion. For this reason we restrict ourselves to computing sensitivity 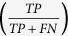 and precision 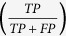 in the results of the evaluation. Sensitivity and precision reported in the following Tables combine the respective values for vertices and edges.

Table 1 summarizes the results of processing the set *I*_1_. The images in *I*_1_ are ideal inputs for NEFI for which all our segmentation routines produce very good results. Otsu’s method and adaptive thresholding yield perfect segmentations. Hence, any difference between NEFI’s output and the ground truth cannot originate in the segmentation part of the pipeline but must be attributed to thinning and graph detection. The excellent correspondence between NEFI’s output and the ground truth confirms that thinning and graph detection are very reliable.

One might question the validity of using graphs which were detected by NEFI in the first place as the input set. However, the approach is valid because the origin of the images in *I*_1_ has no significance regarding NEFI’s performance. In other words, they are just as hard or as easy to process as images obtained in any other comparable way.

The perfect images in *I*_1_ do not represent real life input very well. Therefore we produced three more test sets and evaluate them as described above.

For the set *I*_2_ we take the images in *I*_1_ and change the brightness of the edge drawings randomly. As a result the local contrast between foreground and background varies widely across the image. To create set *I*_3_ we take the images in *I*_1_ and insert a color gradient into the background while leaving the foreground unchanged. Set *I*_4_ is obtained by taking the images in *I*_1_ and subjecting them to a global blur.

Table 2 summarizes the results of processing the set *I*_2_. We observe that both similarity score as well sensitivity are deteriorating for almost all methods except for adaptive thresholding and watershed based on adaptive thresholding. Adaptive thresholding is still able to compensate the local changes in brightness present in the test images and returns segmented images of high quality. We stress that for images showing more severe irregularities the performance of these methods is expected to suffer.

Note that the precision remains comparably high, which indicates that the vertices and edges detected by NEFI are indeed part of the ground truth.

Table 3 summarizes the results of processing the set *I*_3_. We observe that almost all methods, with the exception of adaptive thresholding and watershed based on adaptive thresholding perform very poorly. In particular watershed based on a distance transform marker is completely unable to handle the input images.

Table 4 summarizes the results of processing the set *I*_4_. We observe that almost all methods, with the exception of watershed with distance transform, perform reasonably well. Sensitivity and similarity scores are slightly smaller than the results obtained for the optimal test images *I*_1_. This is due to the fact that blurring an image *i* causes the depicted edges to appear slightly wider. This increase in width is detected in the edges of the graphs *B*_*i*_. As a result the similarity score 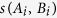 decreases accordingly.

Summarizing the results we conclude that the quality of a graph detected by NEFI depends on the input image and the selected pipeline. We have established that the major factor determining the quality of the extracted graph is indeed the segmentation step. Errors introduced by thinning and graph detection appear negligible in comparison.

Consequently, NEFI’s major limitations arises from the limited applicability of its the segmentation algorithms. As a result, NEFI operates best on clean and uncluttered images such as images produced under controlled laboratory conditions. More difficult input may still be processed, possibly at the cost of reduced quality. For these inputs, domain specific algorithms might be necessary and can be implemented as extensions for NEFI. Alternatively, the segmentation step can be entirely outsourced to more specialized third-party software. Given the externally segmented image as an input, NEFI’s pipeline may proceed directly with graph detection.

We refer the reader to the Supplementary Material for a short guide that summarizes our experience when dealing with more challenging input.

## Evaluation of Speed Performance

NEFI was designed to efficiently process large quantities of images. Thus it outsources computationally intensive tasks to highly optimized and reliable libraries such as Itseez, OpenCV, (2015)^10^, and NetworkX Developer Team, NetworkX, (2014)^23^. Table 5 illustrates the effectiveness of some of NEFI’s algorithms.

## Synergies With Other Software

### Analysis of Graphs

NEFI is a tool that facilitates data acquisition, which is a necessary precursor to data analysis. To analyze NEFI’s output one can either rely on open source graph analysis software^11^,^12^,^13^,^14^,^15^,^16^ or write custom programs. NEFI can output many common graph formats, readable by most popular graph libraries. To get the user started immediately, we provide a minimal Python program that illustrates the basic steps required to perform graph analysis. It shows how to read NEFI’s output from disk and how to compute a histogram of a given edge attribute. The code can be downloaded from NEFI’s project page at the Max Planck Institute for Informatics, http://nefi.mpi-inf.mpg.de, (2015).

### Third-party Segmentation Software

NEFI’s graph detection takes a segmented image as an input. Such an image need not be produced by using NEFI but can be obtained by relying on arbitrary third-party segmentation algorithms or tools.

In this context an interesting tool called ilastik^24^ was brought to our attention. ilastik offers a so-called *classification workflow* in which a pixel classifier is trained by interactive user inputs. The trained classifier can then be used to automatically segment previously unseen images. The segmented images obtained in this way can then directly be turned into graphs using NEFI.

By using NEFI in conjunction with third-party software the benefits of both can be realized.

### General Information about NEFI

NEFI is an open source Python application and available at its project page at Max Planck Institute for Informatics, http://nefi.mpi-inf.mpg.de, (2015). NEFI’s homepage includes a gallery of various use-cases and a comprehensive guide containing instructions on how to download, install and use NEFI on Windows, Mac and Linux. Additionally, a supplementary datasets is available for download there, allowing for a quick evaluation of NEFI’s main features. This dataset can be used to reproduce the figures and evaluation results shown in this manuscript.

## Discussion

We anticipate NEFI to become a valuable tool that allows scientists from any domain to automate graph extraction from images in an intuitive fashion requiring no expert knowledge. We hope that researchers will be able to spend more time on analyzing their data and less time on processing it. By providing a flexible platform for graph extraction, we invite experts to extend and improve NEFI in order to introduce their contributions to a wider interdisciplinary audience. In the long run we would like NEFI to further the field of network science by promoting the creation of new network databases.

## Additional Information

**How to cite this article**: Dirnberger, M. *et al.* NEFI: Network Extraction From Images. *Sci. Rep.* **5**, 15669; doi: 10.1038/srep15669 (2015).

## Supplementary Material

Supplementary Information

## Figures and Tables

**Table 1:** Summary of the evaluation of 250 ideal test images *I*_1_.

Method	Similarity score *s*	Sensitivity	Precision
Otsu’s method	0.984 ± 0.005	0.970 ± 0.011	0.998 ± 0.001
Adaptive threshold	0.984 ± 0.005	0.970 ± 0.011	0.997 ± 0.001
Watershed (deletion/erosion)	0.980 ± 0.006	0.959 ± 0.013	0.988 ± 0.001
Watershed (distance transform)	0.906 ± 0.121	0.837 ± 0.160	0.998 ± 0.001
Watershed (adaptive)	0.977 ± 0.008	0.956 ± 0.016	0.998 ± 0.001
Grabcut (deletion/erosion)	0.984 ± 0.005	0.970 ± 0.011	0.998 ± 0.001
Grabcut (distance transform)	0.983 ± 0.005	0.967 ± 0.011	0.998 ± 0.001

**Table 2:** Summary of the evaluation of 250 test images *I*_2_ with edges drawn with random brightness.

Method	Similarity score *s*	Sensitivity	Precision
Otsu’s method	0.868 ± 0.018	0.704 ± 0.028	0.987 ± 0.005
Adaptive threshold	0.941 ± 0.010	0.853 ± 0.034	0.976 ± 0.025
Watershed (deletion/erosion)	0.859 ± 0.018	0.693 ± 0.028	0.984 ± 0.006
Watershed (distance transform)	0.408 ± 0.176	0.239 ± 0.154	0.987 ± 0.007
Watershed (adaptive)	0.966 ± 0.008	0.936 ± 0.016	0.984 ± 0.017
Grabcut (deletion/erosion)	0.864 ± 0.019	0.696 ± 0.029	0.986 ± 0.005
Grabcut (distance transform)	0.858 ± 0.020	0.688 ± 0.030	0.986 ± 0.005

**Table 3:** Summary of the evaluation of 250 test images *I*_3_ with a color gradient in the background.

Method	Similarity score *s*	Sensitivity	Precision
Otsu’s method	0.737 ± 0.060	0.602 ± 0.065	0.911 ± 0.065
Adaptive threshold	0.984 ± 0.005	0.970 ± 0.011	0.998 ± 0.001
Watershed (deletion/erosion)	0.752 ± 0.047	0.588 ± 0.061	0.977 ± 0.009
Watershed (distance transform)	0.334 ± 0.303	0.240 ± 0.261	0.943 ± 0.043
Watershed (adaptive)	0.982 ± 0.005	0.967 ± 0.012	0.997 ± 0.001
Grabcut (deletion/erosion)	0.733 ± 0.053	0.573 ± 0.066	0.987 ± 0.005
Grabcut (distance transform)	0.742 ± 0.052	0.582 ± 0.065	0.983 ± 0.008

**Table 4:** Summary of the evaluation of 250 blurred test images *I*_4_.

Method	Similarity score *s*	Sensitivity	Precision
Otsu’s method	0.953 ± 0.011	0.909 ± 0.022	0.993 ± 0.003
Adaptive threshold	0.947 ± 0.010	0.863 ± 0.028	0.989 ± 0.005
Watershed (deletion/erosion)	0.950 ± 0.010	0.915 ± 0.022	0.981 ± 0.010
Watershed (distance transform)	0.738 ± 0.127	0.575 ± 0.147	0.915 ± 0.059
Watershed (adaptive)	0.954 ± 0.010	0.909 ± 0.329	0.975 ± 0.020
Grabcut (deletion/erosion)	0.950 ± 0.012	0.903 ± 0.024	0.993 ± 0.003
Grabcut (distance transform)	0.918 ± 0.024	0.838 ± 0.046	0.990 ± 0.004

**Table 5:** Timings of some of NEFI’s pipeline elements on images of different size.

Pipeline element	Image small(1152 × 864)	Image large(5760 × 3840)
Watershed	< 1	2
Adaptive threshold	< 1	7
Guo-Hall thinning	< 1	12
Vertex detection	< 1	5
Edge detection	< 1	6
Computing edge weights	< 1	5

All values are in seconds. The timings were obtained on a Macbook Pro notebook equipped with a 2.4 GHz Intel i5 processor and 8 GB RAM.
