# Supplementary material for: NEFI: Network Extraction From Images
Source: Sci Rep. 2015 Nov 2;5:15669. doi: 10.1038/srep15669 (PMC4629128; doi:10.1038/srep15669)
Supplement: Supplementary Information [file srep15669-s1.pdf]

NEFI: Network Extraction From Images

M. Dirnberger*, T. Kehl and A. Neumann
Max Planck Institute for Informatics
66125 Saarbrücken, Germany

May 20, 2015

Supplementary Information

This document contains information supplementing the main manuscript of *NEFI: Network Extraction From Images*. Complete instructions on how to reproduce the data and images presented in the paper can be found in the download section of NEFI's project page at the Max Planck Institute for Informatics, http://nefi.mpi-inf.mpg.de, (2015).

Methods

Similarity Measure for Evaluation

In order to determine the overall quality of NEFI's output we need to quantify the degree of similarity between two graphs $A = (V_A, E_A)$ and $B = (V_B, E_B)$ using a graph similarity measure. Let $A, B \in \mathcal{G}$, where $\mathcal{G}$ is a set of undirected edges-weighted planar graphs where the nodes are labeled with their respective coordinates in the eucledian plane. Then we may define a similarity measure s as follows:

$$s : \mathcal{G} \times \mathcal{G} \rightarrow [0, 1]$$
$$\forall\ A, B \in \mathcal{G} : s(A, B) = matching(A, B)$$

Where $matching(A, B)$ denotes the normalized cost of a minimum cost graph matching problem which we will define shortly. Given this definition the degree of similarity between A and B is quantified by the value of $s(A, B)$. The similarity measure we propose has the following desirable properties:

- A compared to itself yields a maximum similarity score of $s(A, A) = 1$.
- If there are no vertices in V_A with corresponding similar nodes in V_B and no edges E_A with corresponding similar edges in E_B the minimum similarity score of $s(A, B) = 0$ is obtained.

- The similarity score $s(A, B)$ increases (decreases) if the similarity between A and B increases (decreases).

We proceed with making the notions of *similarity* and *correspondence* more precise by constructing a minimum cost graph matching problem. Given the two graphs $A, B \in \mathcal{G}$, let n_A, n_B denote the number of nodes and m_A, m_B denote the number edges of the respective graphs. Note that n_A and n_B can differ, as can m_A and m_B.

We can now define a matching between A and B consisting of three parts. First we define a matching between the node sets V_A, V_B, then we define a matching between the edges sets E_A, E_B and finally we introduce a coupling between the two. To obtain a solution to the problem we rephrase the combined matching problem as an integer linear program and solve it using IBM CPLEX. For ease of exposition, however, we discuss the problem using the language of matchings.

For the node matching, we match each node $i \in V_A$ with at most one node $j \in V_B$. We define a variable x_{ij} that is 1 if we match i with j and 0 otherwise. With a match we associate a cost of $\Delta_V(i, j)$. The function $\Delta_V(i, j)$ returns the euclidean distance between node i and node j. If any node u remains unmatched, we assign a penalty of $p_V(u)$ to it. A node can either be penalized or matched. We thus compute a minimum cost node matching, in which it is favorable for nodes in A to be matched with similar nodes in B. We call such a matching pair of similar nodes *corresponding*.

The edge matching proceeds analogously to the node matching. Each edge $e \in E_A$ is matched with at most one edge $f \in E_B$. We denote this match with a decision variable y_{ef} and associate a cost of $\Delta_E(e, f)$ with it. The function $\Delta_E(e, f)$ returns the sum of the differences in edge weights between edge e and edge f. If any edge d remains unmatched, we assign a penalty of $p_E(d)$ to it. An edge can either be penalized or matched. We thus compute a minimum cost edge matching, in which it is favorable for edges in E_A to find a match with a similar edge in E_B. We call such a matching pair of similar edges *corresponding*.

As of yet both matchings are independent of each other. In particular this allows an edge e to be matched with an edge f independently from their respective positions in the plane as long as they have similar weights. A more meaningful matching favors pairing similar edges where the endpoints of both edges are similar to each other, i.e. geographically close. This additional constraint introduces a coupling between node and edge matching. To enforce coupling we add the constraint that an edge $e = (a, b) \in E_A$ can only be matched to an edge $f = (a', b')$ if the respective nodes participate in a matching as well. That is, we require a to be matched to a' and b to be matched to b' or alternatively a to be matched to b' and b to be matched to a'.

By combining node matching, edge matching and the additional coupling constraint we obtain the final minimum cost matching problem. We rephrase the uncoupled matching problem as an integer linear program (ILP) as follows:

$$
\begin{aligned}
minimize: \; f = &\sum_{\substack{i\in V_A\\ j\in V_B}} \Delta_V(i,j)x_{ij} \quad + \sum_{i\in V_A} p_V(i)\overline{x}_i + \sum_{j\in V_B} p_V(j)\overline{x}_j \; + \\
&\sum_{\substack{e\in E_A\\ f\in E_B}} \Delta_E(e,f)y_{ef} + \sum_{e\in E_A} p_E(e)\overline{y}_e + \sum_{f\in E_B} p_E(f)\overline{y}_f
\end{aligned}
$$

$$
\begin{aligned}
s.t.: \quad & x_{ij} \in \{0,1\} \qquad i \in V_A \qquad\qquad j \in V_B \\
& y_{ef} \in \{0,1\} \qquad e \in E_A \qquad\qquad f \in E_B \\
& \forall\, i \sum_j x_{ij} \leq 1 \qquad\qquad \forall\, j \sum_i x_{ij} \leq 1 \\
& \forall\, e \sum_f y_{ef} \leq 1 \qquad\qquad \forall\, f \sum_e y_{ef} \leq 1 \\
& \overline{x}_i = 1 - \sum_{j\in V_B} x_{ij} \qquad\qquad \overline{x}_j = 1 - \sum_{i\in V_A} x_{ij} \\
& \overline{y}_e = 1 - \sum_{f\in E_B} y_{ef} \qquad\qquad \overline{y}_f = 1 - \sum_{e\in E_A} y_{ef}
\end{aligned}
$$

To introduce the coupling between the vertex and the edge matching, we add an additional constraint as described above:

$$
\forall\; e = (a,b), f = (a',b'): \quad 2y_{ef} \leq x_{aa'} + x_{ab'} + x_{ba'} + x_{bb'}
$$

Given the optimal solution to the ILP the optimal matching for nodes and edges can be recovered. Thus, we know what matching pairs have been formed and which nodes and edges remained unmatched. We use this information to define true positives, false positives and false negatives as described in the main manuscript.

In addition, we obtain the optimal value of the objective function OPT, i.e. the cost associated with the selected optimal matching. The value of OPT includes both the costs incurred by node and edge matches as well as the penalty terms arising from nodes and edges that cannot be matched. As a result it reflects the similarity between the two graphs A and B.

We point out that $OPT = 0$ if a graph G is matched with itself. Every single node and edge finds its exact copy as a match of cost 0 for itself and thus no penalties arise. The other extreme is realized when there are no vertices in V_A with corresponding similar nodes in V_B and no edges E_A with corresponding similar edges in E_B. In this case the value of OPT attains its maximum $\overline{OPT}$ because all nodes and edges of both graphs are penalized. Naturally, the value of OPT increases (decreases) with decreasing (increasing) graph similarity. Given these observations it is natural to use OPT and $\overline{OPT}$ to define a similarity

measure betweens two graphs $A, B \in \mathcal{G}$ as follows

$$s(A,B) = matching(A,B) = 1 - \frac{OPT(A,B)}{\overline{OPT}(A,B)} \tag{1}$$

with

$$\overline{OPT}(A,B) = \sum_{i \in V_A} p_V(i) + \sum_{j \in V_B} p_V(j) + \sum_{e \in E_A} p_E(e) + \sum_{f \in E_B} p_E(f) \tag{2}$$

As a practical remark, we note that the number of variables entering the integer linear program grows like $\mathcal{O}(n_A n_B + m_A m_B)$. As a result, the linear program may become prohibitively large even for graphs of moderate size.

To meet this issue, it is convenient to define a circle with search radius r centered around each node i. Our goal is to exclusively consider nodes j within this search radius to consider as possible matches for node i. In other words, we label all nodes j outside the search circle as too expensive. To correctly enforce this idea in the integer linear program, we require $p_V(u) > r$ for all nodes u in both graphs.

With this definition in place, none of the nodes j outside of the search circle will be selected as an optimal match for node i because two nodes i and j are matched if and only if the cost of forming the pair is smaller than the penalty due for payment in case the match is not established. Thus, the respective variables x_{ij} may safely be omitted from the linear program.

Choosing the search radius and adjusting the penalties accordingly in order to control the size of the linear program seems more natural to the authors than the other way around. This approach has the additional advantage that a sensible value for the search radius can often be inferred by the looking at the length scales of the structures of interest in the original input image.

The number of variables y_{ef} referring to edge pairs can be reduced by separately applying the previous argument to each of the nodes involved. We enumerate the set of all possible edges $f = (a', b')$ to be matched with edge $e = (a, b)$. The edges $f = (a', b')$ are constructed by connecting all nodes a' within a radius r around the node a with all nodes b' within a radius r around node b. This process can create self-loops $f = (a', a')$ which we exclude as candidates. If there are no nodes within a distance r from a or b, the associated edge variables y_{ef} can be omitted from the linear program. To correctly enforce this idea in the ILP, we normalize the cost of the edges associated with the variables y_{ef} and choose the edge penalties as $p_E(d) > r$ for all edges d in both graphs. There is considerable freedom in the choice of the edge penalties as long as they are large enough that sensible matches y_{ef} are not ignored in favor of paying cheaper penalties.

It is advised to experiment with different values or to simply choose them larger than the largest edge match cost. The latter choice has the effect that whenever a match is available it will be selected, even if the cost is extremely large. As a result this choice is justified in particular when the edge costs are

known to be comparable in magnitude. As a concluding remark, we stress that the choice of penalties for nodes and edges affects the final value of the similarity measure $s(A, B)$ via the normalization. As a result, when using this measure to compare several graphs with each other, the penalties and tracking radius r must be chosen consistently in order to guarantee the viability of the comparison.

Guide on how to use NEFI

Properties of ideal and non-ideal images

In order to obtain good graph extraction results, NEFI relies on input of certain quality. In this section we give a description of desirable properties of ideal input images.

Since, the determining factor of the quality of NEFI's graph extraction is the segmentation step, ideal images should enable a nearly perfect separation of foreground and background. Such images should have a high contrast between the depicted structures of interest and the background. At the same time it is very important that images are free of strong reflections or shadows because such areas are likely to show an even higher contrast to the background than the actual structures of interest. As a result, the segmentation algorithms are prone to identify these regions as foreground causing the actual structures of interest to be ignored. The presence of strong color or brightness gradients can have similar detrimental effects and should thus be prevented if possible. See supplementary Fig. S1 and Supplementary Fig. S2 for examples of challenging images which NEFI will have difficulties working with.

Another factor that influences segmentation, and by extension graph detection, are contaminations of different origin. Parts of the image might not belong to the object of interest at all. Such regions should be removed before loading the image into NEFI, see supplementary Fig. S3.

Supplementary Fig. S4 depicts an image lacking in a similar way. The image contains objects that are technically part of the network one would be interested to extract, however, they are not suited very well to be represented as a graph. In particular, they will be picked up correctly in the segmentation step yielding four large areas of white pixels. Subsequently, thinning will try to reduce these to a lines resulting in more or less unpredictable results. While the remaining parts of the structure will be processed correctly, such images do not constitute ideal candidates for processing with NEFI.

Summarizing this information we can collect the following desirable properties for an ideal image:

- High contrast between foreground and background.
- Uniform background void of reflections, shadows as well as color or brightness gradients.
- No contaminations that might disrupt the segmentation process.

When producing images to be processed with NEFI one should strive to fulfill these properties whenever possible. See supplementary Fig. S5 and supplementary Fig. S6 for examples of very good input images.

How to deal with challenging images

NEFI operates best on images produced under controlled laboratory conditions that fulfill the properties described in the last section. However, more difficult inputs may still be processed, but most likely at the cost of reduced quality. Based on our experience when dealing with more challenging input and the results of our evaluation, we are able to formulate the following recommendations for the usage of NEFI:

Otsu's method may be used on noisy or blurred images. It will do reasonably well as long as if the image has a high contrast between network and background. Adaptive threshold, watershed based on adaptive threshold, and GrabCut with deletion and erosion may even perform slightly better under these conditions. Otsu's has the advantage that no parameters have to be set in order to get good results. The choice should be based on the desired degree of resolution of the extracted graph. We would recommend these methods to process supplementary Fig. S3 after clipping the image accordingly.

Adaptive threshold and watershed based on adaptive threshold may be used if differences in contrast between foreground and background are local and not too strong. If this is the case good results may still be obtained. Both methods allow to analyze images that contain a color gradient in the background or that contain edges which have differing levels in brightness. One might try to process images like supplementary Fig. S1 and supplementary Fig. S2 with these methods while experimenting with different parameter settings. However, such images remain challenging. NEFI's current algorithms may not deliver sufficient results.

Preprocessing methods like Gaussian and Median Blurring, Denoising as well as Bilateral Filtering can be used to remove small artifacts, contaminations or irregularities from the image. Although these methods can reduce the amount of artifacts produced during segmentation and thinning, improvements come at a price. For example, imposing a strong blur causes the depicted edges to appear slightly wider. This effect will propagate through the pipeline causing the edge widths of the final graph to overestimate the true widths depicted in the image. We recommend to use preprocessing with care especially if a high accuracy regarding edge weights is required.

Graph filtering enables the removal of unwanted artifacts and spurious vertices caused by irregularities in the input which propagate through the pipeline. However, filtering can only repair the result up to a given point.

While the ability to add custom filters is powerful, it appears pointless to filter a graph established on the basis of a failed segmentation. We encourage users to visually verify the integrity of the established graph and only then to proceed with filtering.

We anticipate users to encounter images NEFI's algorithms will be unable to handle properly. In this situation the user will have to rely on different segmentation solutions.

As a first suggestion, we recommend to user to look for available software specializing in segmentation. In this regard we like to point out the Kitware, ITK Project, (2015), [1]. It's *C++* code base has been developed by the medical image processing community and serves as the basis for many other specialized tools that deal with image processing as well as segmentation.

After a segmented image has been obtain using specialized third party segmentation software, NEFI can take this image and proceed with graph detection and filtering directly.

If no proper software is available, the user has the option to extend NEFI's segmentation capabilities by adding more sophisticated code. When doing so, one can built on top of existing features already implemented in NEFI. The literature offers a wealth of different approaches to segmentation leading to algorithms of varying complexity. Before diving into any implementation efforts, we strongly recommend to survey existing methods and their domains of effectiveness by consulting [2–4].

References

1. Johnson, H. J., McCormick, M., Ibáñez, L. & Consortium, T. I. S. *The ITK Software Guide* Third. *In press.* Kitware, Inc. (2013).
2. Chellappa, R. & Manjunath, B. English. in *Foundations of Image Understanding* (ed Davis, L.) 219–240 (Springer US, 2001).
3. Pal, N. R. & Pal, S. K. A review on image segmentation techniques. *Pattern Recognition* **26,** 1277 –1294 (1993).
4. Pham, D. L., Xu, C. & Prince, J. L. Current methods in medical image segmentation 1. *Annual review of biomedical engineering* **2,** 315–337 (2000).

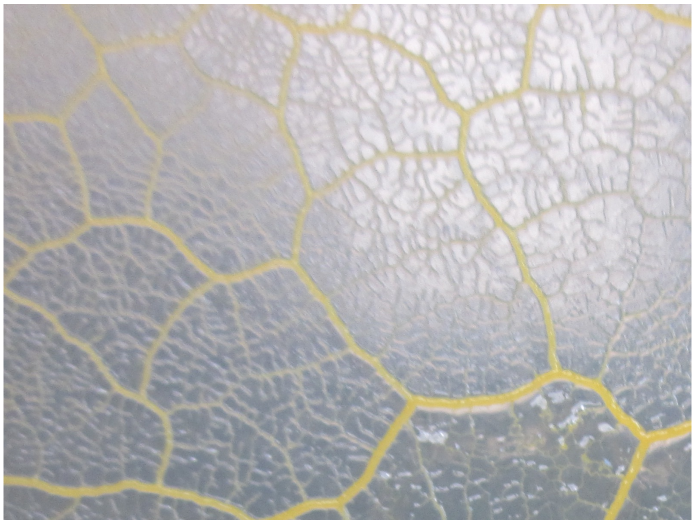

Supplementary Fig. S1: This image of *P. polycephalum* contains strong light reflections in the upper right quadrant which will cause NEFI's segmentation algorithms to be lead astray. The fact that the image is not properly focused is less of a problem in comparison.

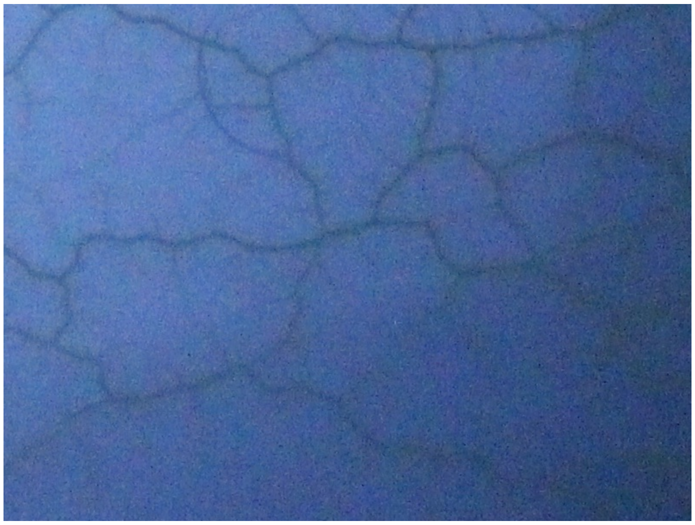

Supplementary Fig. S2: This image of *P. polycephalum* was not illuminated evenly from below. As a result it contains a brightness gradient which is detrimental for many of the segmentation algorithms currently implemented in NEFI. The fact that the image contains a lot of visible noise makes it a bad candidate for processing with NEFI.

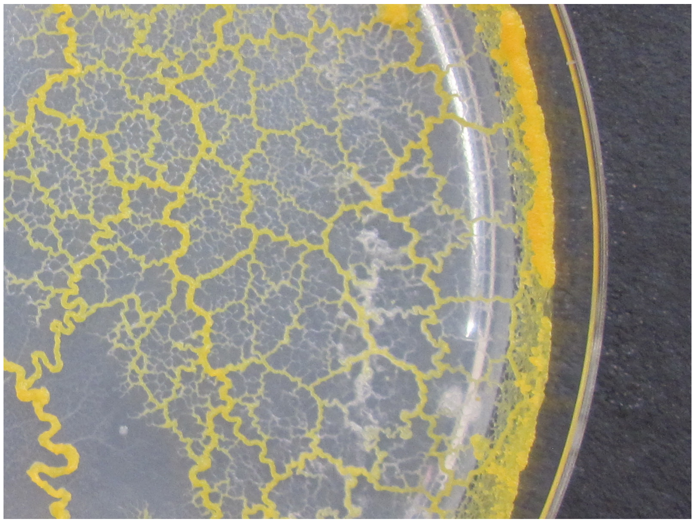

Supplementary Fig. S3: In addition to the network of *P. polycephalum* the image contains the edge of a petri dish and pieces of the background on the right hand side (as well as light reflections). Prior to processing the image, petri dish and background should be removed.

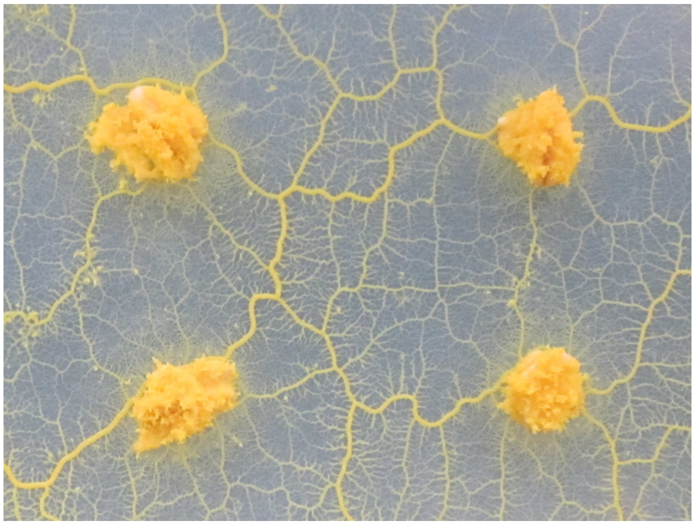

Supplementary Fig. S4: The network of *P. polycephalum* depicted in the image contains 4 massive regions (oat flakes completely covered by the mold). After segmentation, thinning will try to reduce these regions to lines leading to unpredictable results. The remaining parts of the network, however, will be extracted correctly.

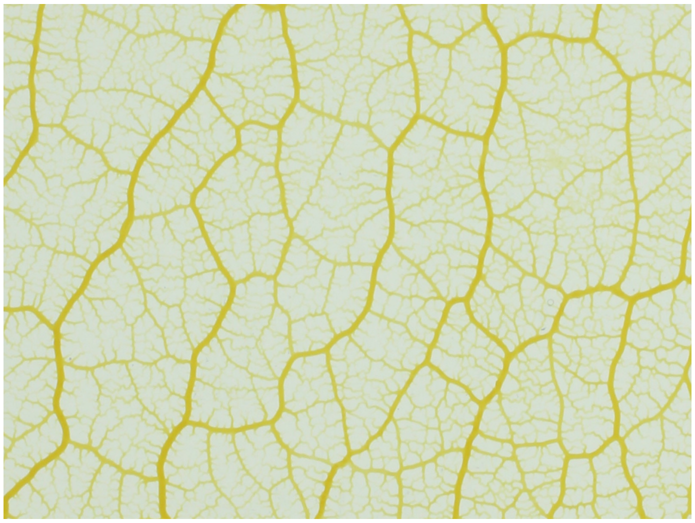

Supplementary Fig. S5: An ideal image of *P. polycephalum*. The image has been obtained using bright field illumination and was produced in a collaboration with the KIST Europe.

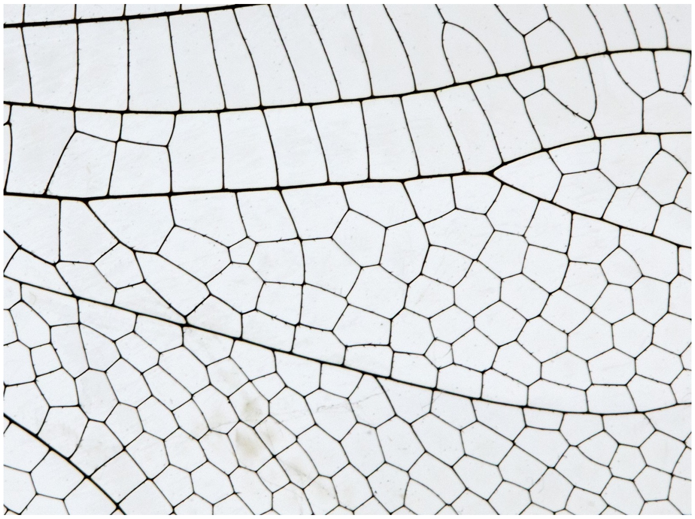

Supplementary Fig. S6: An ideal image depicting a detail of the wing of *A. junius*. Image courtesy of Pam and Richard Winegar.

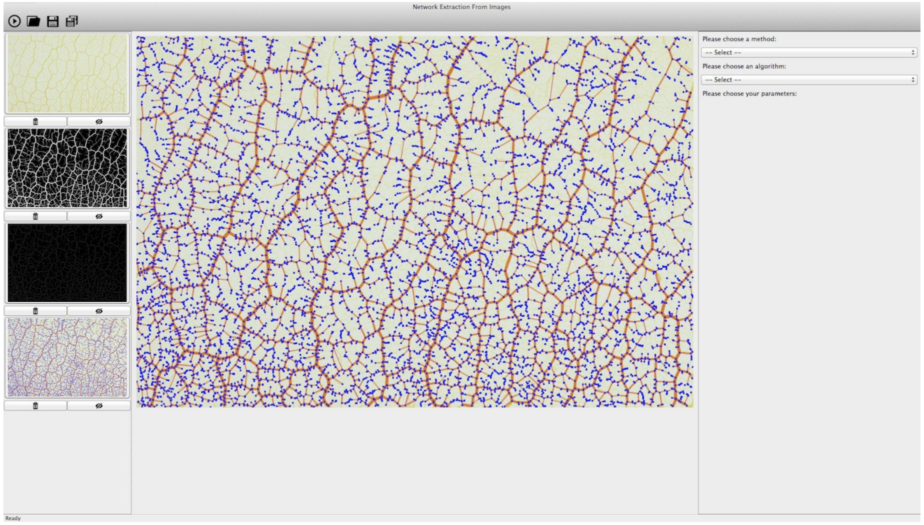

Supplementary Fig. S7: A screenshot of NEFI's GUI running on Mac OS. On the left hand side NEFI lists intermediate results as thumbnails. Bringing the final result to the center workspace allows for direct visual assessment of the quality of the extracted graph. On the right hand side NEFI's pipeline elements can be accessed via drop-down menus. The image was produced in a collaboration with the KIST Europe.
